# Variation in Actionable Pharmacogenetic Markers in Natives and Mestizos From Mexico

**DOI:** 10.3389/fphar.2019.01169

**Published:** 2019-10-10

**Authors:** Vanessa Gonzalez-Covarrubias, Marlet Morales-Franco, Omar F. Cruz-Correa, Angélica Martínez-Hernández, Humberto García-Ortíz, Francisco Barajas-Olmos, Alma Delia Genis-Mendoza, José Jaime Martínez-Magaña, Humberto Nicolini, Lorena Orozco, Xavier Soberón

**Affiliations:** ^1^Pharmacogenomics Laboratory, INMEGEN, CDMX, Mexico City, Mexico; ^2^Immunogenomics and Metabolic Diseases Laboratory, INMEGEN, CDMX, Mexico City, Mexico; ^3^Genomics of Psychiatric and Neurodegenerative Diseases Laboratory, INMEGEN, Mexico City, Mexico

**Keywords:** pharmacogenomics, population variation, next generation sequencing, pharmacodynamics, pharmacokinetics

## Abstract

The identification and characterization of pharmacogenetic variants in Latin American populations is still an ongoing endeavor. Here, we investigated SNVs on genes listed by the Pharmacogenomics Knowledge Base in 1284 Mestizos and 94 Natives from Mexico. Five institutional cohorts with NGS data were retrieved from different research projects at INMEGEN, sequencing files were filtered for 55 pharmacogenes present in all cohorts to identify novel and known variation. Bioinformatic tools VEP, PROVEAN, and FATHMM were used to assess, *in silico*, the functional impact of this variation. Next, we focused on 17 genes with actionable variants that have been clinically implemented. Allele frequencies were compared with major continental groups and differences discussed in the scope of a pharmacogenomic impact. We observed a wide genetic variability for known and novel SNVs, the largest variation was on *UGT1A > ACE > COMT > ABCB1* and the lowest on *APOE* and *NAT2*. Although with allele frequencies around 1%, novel variation was observed in 16 of 17 PGKB genes. In Natives we identified 59 variants and 58 in Mestizos. Several genes did not show novel variation, on *CYP2B6*, *CYP2D6*, and *CYP3A4* in Natives; and *APOE*, *UGT1A*, and *VKORC1* in Mestizos. Similarities in allele frequency, comparing major continental groups for VIP pharmacogenes, hint towards a comparable PGx for drugs metabolized by *UGT1A1*, *DPYD*, *ABCB1*, *CBR3*, *COMT*, and *TPMT*; in contrast to variants on *CYP3A5* and *CYP2B6* for which significant MAF differences were identified. Our observations offer some discernment into the extent of pharmacogenetic variation registered up-to-date in Mexicans and contribute to quantitatively dissect actionable pharmacogenetic variants in Natives and Mestizos.

## Introduction

Pharmacogenetic studies in Mexican populations have been directed towards the identification of markers previously reported with functional consequences for drug safety and efficacy (Contreras et al., 2011; Bonifaz-Pena et al., 2014; Marsh et al., 2015; Fricke-Galindo et al., 2016). The advent of faster and more accessible technologies has made feasible to investigate a broader swath of the pharmacogenome including the presence of novel variants. In most of the developed world pharmacogenetic testing is being implemented with the aid of consortia and institutions such as the PGRN, the PGKB, CPIC, and the FDA. These offer a list of genetic variants to guide drug selection, dose optimization, and to reduce the risk of adverse drug reactions. Moreover, implementation programs such as The electronic Medical Records and Genomics (eMERGE) initiative have started preemptive pharmacogenomics testing in over 10,000 patients, on the basis of the benefits of pharmacogenetic information, despite the so-called lack of cost–benefit evidence (van Driest et al., 2014). Nevertheless, in developing countries broad pharmacogenetic implementation is even more incipient, a curated collection of local variants has not been yet defined, and for many relevant markers there are significant differences for minor allele frequencies (MAF) when comparing to major continental groups. For example, Campos et al. reported lower CYP2C19*17 rs1224856 allele frequencies in 346 Mexican Americans compared to CEU, MAF: 0.14 vs. 0.22, which may be indicative of lower frequency of bleeding with clopidogrel in the former (Claudio-Campos et al., 2015). In Mexicans higher allele frequencies have been reported for *CYP3A4* rs2750574 and *NQO1* rs1800566, which may be differentially affecting the efficacy of tacrolimus, fluorouracil, and anthracyclines, when compared to Europeans.

Mexico is home to 68 genetically different ethnic groups which suggests urgency for an adequate collection, classification, and characterization of variants on genes that affect drug efficacy and safety. Published studies collect the identification and determination of allele frequencies of at least all PGKB level 1 variants in Mestizos (Cuautle-Rodriguez et al., 2014; Fricke-Galindo et al., 2014; Fricke-Galindo et al., 2016; Gonzalez-Covarrubias et al., 2017). Fewer studies have included Natives, NGs techniques, or genotype-phenotype assessments. Results accede that for several makers, allele frequencies and its clinical impact differ among Natives, Mestizos and, major continental groups (Bonifaz-Pena et al., 2014; Fricke-Galindo et al., 2016; de Andrés et al., 2017) In addition, little is known of the presence and frequency of local private variation, which can only be investigated though sequencing (Moreno-Estrada et al., 2014). NGS studies have also shown that it is the interplay of several variants, rather than one maker, that explains drug response variability (Johnson et al., 2011; Beekman et al., 2013; Katsila and Patrinos, 2015; Yang et al., 2016). In this regard, Han S.M. et al. identified dozens of rare variants with functional consequences to drug response, but more importantly, it revealed that targeted sequencing enabled profiling of actionable and rare variants, some of yet unknown functional consequences (Han et al., 2017). Furthermore, *in vitro* studies of major pharmacogenes have confirmed that many rare variants do have a functional impact confirming NGS bioinformatic predictions (Matimba et al., 2009; Han et al., 2017). More limited in scope, our own work has documented the importance of variants, derived from NGS, to increase the predictive value of genotypes on the pharmacokinetics of atorvastatin (Cruz-Correa et al., 2017) and the pharmacodynamics of cumarins (Gonzalez-Covarrubias et al., 2017). Nevertheless, the collection of a comprehensive pharmacogenetic set of variation is still underway, mostly for understudied populations.

Here, we identified, and classified pharmacogene variation on 17 genes listed by the PGKB in 1284 Mestizos and 94 Natives from several institutional cohorts at the National Institute for Genomic Medicine in Mexico, from which NGS data were available. Allele frequency comparisons showed that pharmacogenetic variation is significantly different between Natives and Mestizos for several PGKB variants. We observed twice as many actionable variants in Mestizos vs. Natives, but 4× more novel variation *per* individual in Natives suggesting that the pharmacogenome in the latter is far from complete, and that current pharmacogenetic guidelines may prove to be more beneficial for certain populations within the country.

## Methods

**Participants and Sequencing Files**. We analyzed WES data from 1,284 Mexican Mestizos published at the ExAC platform (Lek et al., 2016; Karczewski et al., 2017) and three institutional cohorts sequenced with custom probes using different Haloplex v2, Agilent Technologies protocols, thus variant calling was performed independently for each cohort. In addition, sequencing files for 94 Natives were procured by the 100 Genome Consortium (INMEGEN, to be published) and the project Metabolic Analysis in an Indigenous Sample (MAIS) (Contreras-Cubas et al., 2016). All participants provided a blood sample after signing an informed consent and all research projects were approved by the Ethics and Research Committees at INMEGEN. Participants were self-reported as Natives and confirmed by AIMS analyses (to be published). DNA from Natives was sequenced by BGISEQ-500 (Cambridge, MA, USA). Samples from Natives belonged to 36 ethnic groups thus, further stratification and multiple comparisons within Natives or with Mestizos was not possible. Similarly, for Mestizos >90% of all DNA samples came from individuals from the center of the country limiting further stratification. In summary, vcf data came from five institutional cohorts: WGS (Natives = 94, to be published), WES (Mestizos = 968) (Flannick et al., 2019), NGS-targeted 1 (Mestizos = 110) (Gonzalez-Covarrubias et al., 2017), NGS-targeted 12 (Mestizos = 146) (Gonzalez-Covarrubias et al., 2016), and NGS-targeted 3 (Mestizos = 60) (Cruz-Correa et al., 2017). All data were processed according to the Broad Institute recommended best practices workflow and the Genome Analysis ToolKit (GATK) (Auwera et al., 2013).

To assess the functional impact of variants in coding and non-coding regions, we selected three algorithms. We utilized PROVEAN, VEP, and FATHMM which are well published and have been used by the scientific community (Devarajan et al., 2019). PROVEAN predicts whether a SNV affects the function of a protein by giving a score based on sequence alignment; the lower the score, the more dissimilar is the substitution compared to the reference (Mizzi et al., 2014; Wendt et al., 2018). VEP (Variant Effect Predictor) seeks for genes/transcripts of a variant to determine its effect at the amino acid level (e.g. stop gained, missense, stop lost, altered splicing, frameshift, stop loss, and start loss), a high impact predicted by VEP is considered as deleterious (Oetting et al., 2016; Lakiotaki et al., 2017). FATHMM was utilized to predict functional consequences of non-coding variants, it integrates functional annotations from ENCODE and calculates a score (0–1). Scores >0.5 are indicative of deleterious variation (McInnes et al., 2019; Zhou et al., 2018), and although non-coding variants may not have a direct impact on a protein the overall regulation may render faulty. These tools have been utilized in PGx research (Devarajan et al., 2019), and we have reported their use to filter variants with potential effects on atorvastatin pharmacokinetics with acceptable results. PGx-oriented algorithms for the prediction of the functional impact of novel variants are being developed and validated, these new tools include some of the algorithms utilized here (Zhou et al., 2018; Zhou et al., 2019).

Genotypes of PGKB variants in **Table 2** were in complete concordance with previous genotyping experiments by RTPCR ex. *CYP2C9/VKORC1* (Villegas-Torres et al., 2015), DMET microarray (Gonzalez-Covarrubias et al., 2016) and by NGS (Gonzalez-Covarrubias et al., 2017).

***Pharmacogenetic Analyses***. Statistical analyses focused on 55 genes since these were identified in all samples. In-depth analyses were undertaken for variants listed by the PharmGKB with a level of evidence 1 and 2 (Relling and Klein, 2011). PGKB level 1 (1A/1B) represent variation with a strong evidence of PK/PD alteration, and these have been published as markers in CPIC dosing guidelines. Level 2 refers to variants with moderate evidence to be linked to a drug’s safety or efficacy. We computed allele frequencies and performed comparisons for several populations, CEU, Northern Europeans from Utah (Caucasians); MXL, Mexicans from Los Angeles; YRI, Yoruba in Ibadan, Nigeria; CHB, Chinese Han from Beijing. Data analyses, descriptive statistics, allele frequency calculations, and variant inferences were performed with R (Team R Core, 2014), PLINK (Purcell et al., 2007), and PGKB resources (Sangkuhl et al., 2008).

## Results

**NGS Summary.** Sequencing files from 94 Natives and 1,284 Mestizos showed a depth-coverage between 50× and 600×, tNGS aimed for 100× coverage while WES and WGS for at least 50×. Depth coverage varied per genomic region regardless of the theoretical depth calculated per sequencing run. For example, tNGS showed a range of depth and coverage of 40×–600× and 80–100% for the selected genes, but after quality control some pharmacogenetic relevant regions including that of *VKORC1* rs9323231 and CYP2C19*17 rs12248560 were not covered.

In Natives, preliminary analyses identified the largest number of variants as data came from WGS, NGS-targeted and WES in Mestizos yielded a lower count. Together, all cohorts gathered 436 pharmacogenes, on which we identified on average 102 novel variants *per* individual in Natives, a value almost three times higher than in Mestizos. Consistently, non-synonymous SNVs (single nucleotide variants) were five times more abundant in Natives, suggesting a genetic diversity uncovered by NGS, but these 436 genes were not identified in all individuals. Therefore, we directed further analyses on genes identified in all cohorts and genes listed by the PGKB with a validation level of 1 or 2, which are the focus of this report.

### Pharmacogenetic Analyses

Fifty-five genes were identified in all in 1,378 individuals including most CYPs, FMOS, members of the *UGT1A* family, *SULT1A*, and 11 genes involved in pharmacodynamics. **Table 1** lists variation for these 55 genes many of which are listed by the eMERGE initiative and the Pharmacogenomics Research Network (Bush et al., 2016). On these, we observed 3.4× and 2.5× more variants in Mestizos vs. Natives (novel and known), as expected given the larger sample of Mestizos. However, the average number of variants per individual was 3.9× and 3.4× higher (novel and known) in Natives, reflecting the larger diversity unreported in the latter. Also, private variants, i.e., polymorphisms observed as heterozygous in only one individual, were more frequent in Natives (n = 868) vs. Mestizos (n = 3256, **Table 1**).

**Table 1:** Variation in 55 pharmacogenes shared among cohorts.^1^

	Natives			Mestizo		
	Novel		All^1^	Novel		All^1^
N, individuals		94			1,284	
Genes		55			55	
Total variants	528		3,092	1,809		7,627
Variants *per* individual	14		818	4		230
Average MAF of variants	0.043		0.117	0.004		0.029
Private variants	349		868	1,090		3,256
Private *per* individual	4		9	1		3
^2^Functional	228		1,511	865		4,224
Deleterious	128		621	280		1,828
Non-synonymous SNV	55		395	134		1,489
Synonymous SNV	80		348	44		861
Multiple AA Change	0		0	14		14
Frameshift	4		13	102		151
Nonsense	0		1	7		54
Others	89		754	557		1,644

^1^All refers to all variants known (rs identifier) and novel. ^2^Functional variants are those with an in silico functional consequence. Pharmacogenes included: ADH1C1, CYP3A43, COMT, UGT1A9, ACE, CBR1, CYP3A5, NAT1, UGT2B7, APOE, CBR3, CYP3A7, NAT2, ABCB1, DRD2, CYP11B1, DPYD, SULT1A1, SLC16A1, F8, CYP11B2, FMO1, TPMT, SLC22A11, F9, CYP1A1, FMO2, UGT1A1, FTO, CYP1A2, FMO3, UGT1A10, LPA, CYP2B6, FMO4, UGT1A3, P2RY2, CYP2C18, FMO5, UGT1A4, TOMM40L, CYP2C19, MAOA, UGT1A5, VDR, CYP2C9, MAOB, UGT1A6, VKORC1, CYP2D6, NQO1, UGT1A7, CYP3A4, UGT1A8, SLCO1B1.

**Figure 1:**
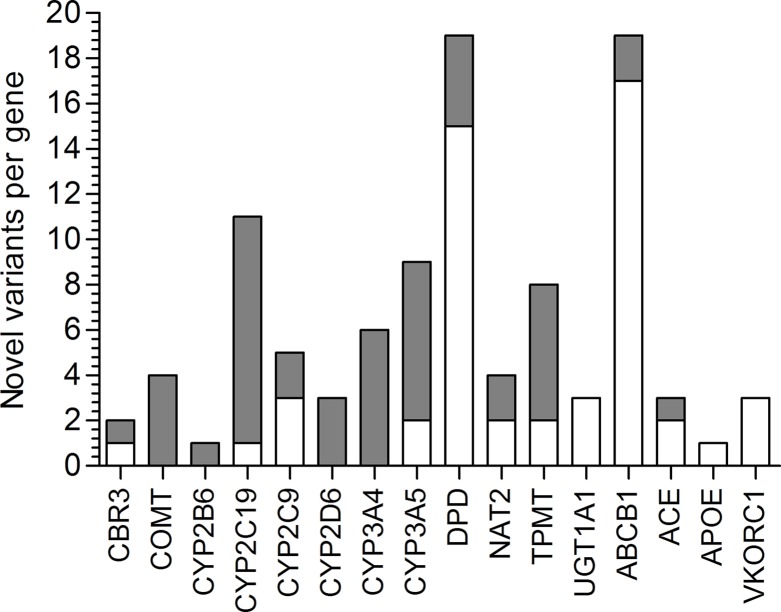
Novel variation and its functional impact identified in 16 PGKB genes in Natives (gray) and Mestizos (blank).

Next, we focused on 17 actionable genes with clinically validated variants to perform comparisons between populations. Genes were selected according to the PGKB classification considering only those with a validation level 1 and 2, included in published CPIC dosing guidelines also, considered actionable by the FDA. These 17 genes were, *ABCB1, ACE, APOE, CBR3, CYP2C19, CYP2C9, CYP2B6, CYP2D6, CYP3A5, CYP3A4, COMT, DPYD, NAT2, SLCO1B1, TPMT, UGT1A*, and *VKORC1*. On these, we observed 2,387 non-private, novel, and known variants, 34 of which were listed by the PGKB. The largest number of known variants were observed on *ACE* (251), *UGT1A* (318), *ABCB1* (182), and *COMT* (235), (***Figure 1***). Genes with the lowest number of variants were *NAT2* (33) and *APOE* (24), again with a higher count in Mestizos.

Next, we determined minor allele frequencies for these 17 PGKB-listed variants and compared them between Mexicans and those reported for major continental populations. In Natives, we observed a higher MAF, compared to Mestizos, for *VKORC1* rs8050894 (1.4×), *CYP2B6* rs2279343 (1.5×), and *CYP3A5* rs776746 (2×), with potential implications for the differential management in Natives for cumarins, efavirenz, nevirapine, methadone, and tacrolimus am ong several others. For most of these variants allele frequencies in Natives were higher compared to Mestizos, also when compared to CEU and CHB (**Table 2**).

**Table 2:** Allele frequencies for 17 actionable pharmacogenetic variants.

Variants	Natives	Mestizo	gMAF^1^	MXL^1^	CEU^1^	YRI^1^	CHB^1^	PGKB, minor allele impact, drugs
**ABCB1 (182)**	66	118						Decreased enzyme activity affecting toxicity and efficacy of nevirapine, ondasteron, methotrexate, fentanyl, digoxin, simvastatin
rs1045642	0.436	0.515	0.395	0.480	0.430	0.880	0.620	
rs2032582	0.410	0.539	0.334	0.550	0.560	1	0.440	
**ACE (251)**	72	273						No PGKB variants detected
**APOE (24)**	6	22						PGKB evidence level 2
rs7412	0	0.036	0.075	0.050	0.070	0.110	0.110	Atorvastatin efficacy
**CBR3 (40)**	10	34						PGKB evidence level 2
rs1056892	0.277	0.241	0.427	0.280	0.310	0.510	0.390	Anthracyclines PK
**CYP2C19 (173)**	62	170						PGKB evidence level 1
rs4244285	0.053	0.1	0.220	0.110	0.130	0.170	0.340	PM, decreased enzyme activity; drugs affected: proton pump inhibitors, clopidogrel, citalopram, imipramine, diazepam, mephenytoin
rs4986893	0	0.001	0.1	0	0	0	0.040	
rs28399504	0	0.006	0.002	0.010	0	0	0.010	
**CYP2C9 (96)**	37	72						PGKB evidence level 1
rs1799853	0.011	0.049	0.050	0.100	0.150	0	0	Poor metabolizers, decreased enzyme activity; drugs affected: NSAIDs, phenytoin, cumarins
rs1057910	0.016	0.031	0.050	0.020	0.070	0	0.040	
**CYP2B6 (103)**	50	89						PGKB evidence level 1 and 2
rs2279343	0.351	0.240	0.302	0.270	0.210	0.460	0.190	Altered enzyme activity; affecting toxicity and pharmacokinetics of efavirenz, nevirapine, methadone.
rs28399499	0	0.003	0.023	0.01	0	0.120	0	
rs3745274	0.351	0.318	0.295	0.31	0.280	0.400	0.160	
rs4803419	0.596	0.522	0.289	0.420	0.280	0.04	0.500	
**CYP2D6 (76)**	38	72						Other relevant PGKB genes
rs1065852	0.124	0.102	0.240	0.150	0.240	0.110	0.400	PM, decreased enzyme activity affecting, loperidone, escitalopram, nevirapine, timolol
rs28371706	0.005	0.007	0.060	0	0	0.750	0	
rs28371725	0	0.028	0.064	0.020	0.120	0.010	0.030	
rs16947	0.796	0.778	0.360	0.740	0.680	0.440	0.840	
**CYP3A5 (90)**	26	88						PGKB evidence level 1 and 2
rs776746	0.303	0.189	0.380	0.230	0.040	0.830	0.310	PM, decreased enzyme activity; drugs affected: tacrolimus.
**CYP3A4 (76)**	29	78						
rs2740574	0.069	0.071	0.230	0.070	0.020	0.210	0	
**COMT (235)**								
rs4680	0.399	0.391	0.370	0.400	0.470	0.310	0.320	Decreased activity affecting nicotine replacement therapy
**DPYD (119)**	48	105						PGKB evidence level 1
rs67376798	0	0.001	0.002	0	0.010	0	0	PM, decreased enzyme activity; affecting fluoropyrimidines
**NAT2 (33)**	15	26						PGKB evidence level 1
rs1041983	0.303	0.299	0.400	0.270	0.300	0.500	0.360	PM, decreased enzyme activity; affecting: ethambutol, isoniazid, pyrazinamide, rifampin
rs1799930	0.059	0.120	0.265	0.130	0.300	0.200	0.300	
**SLCO1B1 (124)**	60	98						PGKB evidence levels 1 and 2
rs4149056	0.117	0.091	0.090	0.080	0.150	0.010	0.140	Impaired transporter activity affecting statins
rs4149015	0	0.017	0.055	0.020	0.040	0	0.120	
**TPMT (82)**	27	54						Other relevant PGKB genes
rs1800462	0	0.002	0.002	0	0	0	0	Poor metabolizers, decreased enzyme activity, affecting tiopurines capecitabine
rs1800460	0.048	0.049	0.010	0.040	0.030	0	0	
rs1142345	0.048	0.052	0.040	0.050	0.030	0.060	0.010	
**UGT1A (318)**	68	268						PGKB evidence levels 1 and 2
rs887829	0.335	0.3	0.350	0.370	0.320	0.520	0.110	Decreased protein levels affecting irinotecan, deferasirox, atazanavir
rs4148323	0.043	0.030	0.030	0.020	0.010	0	0.230	
**VKORC1 (66)**	22	61						PGKB evidence level 1
rs7294	0.441	0.447	0.420	0.35	0.31	0.510	0.040	PM, decreased enzyme activity; drugs affected: cumarins
rs8050894	0.537	0.381	0.420	0.510	0.430	0.230	0.960	
rs9934438	0.537	0.431	0.360	0.470	0.430	0.030	0.960	
rs9923231	0.543	0.402	0.360	0.470	0.430	0.030	0.960	

^1^Data from 1000 Genomes (Auton et al., 2015). In parenthesis, total number of variants identified per gene.

The opposite was also observed, i.e., lower allele frequencies in Natives were identified for *CYP2C19* rs4244285 (2×), *CYP2C9* rs1799853 (4.5×), rs1057910 (2×), *NAT2* rs179930 (2×), *SLCO1B1* rs4149015, and *APOE* rs7412. The latter two were not found in Natives. *CYP2C19* and *CYP2C9* lower allele frequencies in Natives may be indicative of a lower proportion of poor metabolizers potentially affecting the pharmacokinetics of over 18% of all drugs. For the remaining 26 variants on 8 genes, allele frequencies were comparable between Natives and Mestizos. A summary of variation on these 17 genes is presented in **Table 2**.

### Novel Variants on Actionable PGKB Genes

Using NGS data we sought for novel variation, and identified 116 not-previously reported variants on 16 of the 17 PGKB genes (MAF ≥1%). We found some novel variation in all genes studied in Mestizos or Natives except for *SLCO1B1*. The functional impact of these variants was assessed by the algorithms, VEP, Provean, and FATHMM (Dong et al., 2015). **Table 3** lists novel variants that were predicted as deleterious by more than two algorithms, observed on *ABCB1* (4), *CYP2C19* (1), and *CYP3A4* (1), and although their frequency does not exceed 2% we believe their strongly predicted functional impact pinpoints them as worth investigating. A full list of novel variants annotated by the three algorithms is listed in **Supplementary Table 1**.

**Table 3:** Selected novel variants predicted as deleterious.^1^

Gene	Position	Nucleotide	Functional impact	MAF %
ABCB1	7:87165002	C/A	transcript	1.85
ABCB1	7:87160718	C/A	synonymous	1.60
ABCB1	7:87160786	T/A	missense	1.06
ABCB1	7:87179286	A/G	synonymous	1.06
CYP2C19	10:96609809	T/A	missense	1.15
CYP3A4	7:99377662	C/A	missense	1.47

^1^For a full list see **Supplemental Table 2**.

Lack of novel variation was detected for *APOE*, *UGT1A1*, and *VKORC1* in Mestizos, and for *CYP2D6*, *CYP3A4*, *CYP2B6, and COMT* in Natives (**Figure 1**). The largest count of novel variants in Natives was observed for *ABCB1* (25) and *DPYD* (15), and in Mestizos on *CYP2C19* (10) and *CYP3A4*/5 (13). Novel variation was on average comparable between population groups, but differed largely *per* gene **Figure 1** and **Supplementary Table 1**.

## Discussion

As genetic variation is better defined within populations it is relevant for each country to assess whether current pharmacogenetic platforms can offer direct benefits for their people. There are 68 Native groups in Mexico representing around 10% of the population, but admixture proportions vary greatly throughout the country. Thus, we sought to determine and compare differences in gene variation related to pharmacokinetics and pharmacodynamics in Natives and Mestizos by consolidating different institutional cohorts. Sequencing data for all 1,378 DNA samples were obtained, by various groups utilizing different NGS approaches, and our analyses focused on 17 PGKB genes. Despite the sample size difference between Natives (94) and Mestizos (1284) we were able to capture representative pharmacogenetic variation for both groups. Not unexpectedly, Natives showed almost 10× more variants, which hints towards a larger proportion of unaccounted variation (novel and known).

Comparative analyses among Mestizos, Natives, and continental populations, highlight the extent of our incomplete registry of pharmacogenetic variability within the country, but also indicate a closer completion for Mestizos than for Natives, because these have been much less studied (Cid-Soto et al., 2018; Sánchez-Pozos et al., 2018). For instance, Natives showed 2.4 functional novel variants on average per individual compared to 0.7 in Mestizos, which is in agreement with Romero-Hidalgo et al. (2017) showing that damaging variation is 2× higher for unreported variants in Native Americans. These observations indicate that genetics may underlie drug disposition differences not only between Mexico and major continental groups, but also within the country’s populations.

### PGx Markers in Guidelines for Clinical Implementation

CPIC dosing guidelines follow data curation by the PGKB and represent the most useful pharmacogenetic resource around the world. Therefore, local adoption of pharmacogenetics in developing countries requires identifying the frequency of already published actionable markers, and to assess if their presence warrants its application to local populations prior to implementation. Here, we compared allele frequencies for 34 PGKB variants in 17 genes with a validation level 1 and 2 to assess if they were similar or different within the studied populations, and were also compared to the major ancestral population (**Table 2**). First, we discuss variants with higher allele frequency in Natives vs. Mestizos vs. CEU. These were observed on *VKORC1, CYP3A5, and UGT1A1*. Cumarin-sensitivity variants *VKORC1* rs8050894, rs994438, and 9923231, all in LD, were 25–40% more frequent in Natives hinting for higher cumarin sensitivity. However, only 1.6% of Natives showed variants on *CYP2C9* vs. 5–10% of Mestizo and CEU. In Natives *VKORC1* variation may reflect higher sensitivity to cumarins, but the low CYP2C9*2/*3 frequencies a higher metabolism this particular *VKORC1–CYP2C9* interplay may abrogate cumarin dosing adjustment in Natives. It is relevant to consider that this is mostly the case for warfarin since other CYPs such as CYP2C8 are responsible for R-acenocumarol metabolism, the isomer to which the anticoagulant effect is ascribed.

CYP3A5*3, rs776746 affects the disposition of over 20 drugs, but it is particularly relevant for cyclosporine and tacrolimus. In Europeans the T allele has a frequency of 4%, significantly lower than the one observed in Natives (MAF: 0.303) and Mestizos (MAF: 0.189). It is possible that these differences may lead to more dose adjustments for tacrolimus in Mexicans compared to CEU (Zhu et al., 2011), but similar to that in Asians (MAF: 0.311) (Niioka et al., 2012).

Variants on *UGT1A1* significantly influence the pharmacokinetics of several drugs including irinotecan and atazanavir, lower enzyme levels are a consequence of several polymorphisms including, rs887829 and rs4148323, the former showed similar allele frequencies in Natives, Mestizos, MXL, and CEU, but rs4148323 was 4× and 3× more frequent in Natives and Mestizos compared to CEU, this may be indicative of low glucuronidation in a higher proportion of Mexicans. The overall impact of these differences cannot be conclusive, but it is relevant for many other drugs, for which its clearance rate is limited by UGT1A1. Our NGS data did not identify other key variants such as UGT1A1*28 rs3064774 or rs4148323 which have been shown to be differently distributed in other Latin American countries (Marsh et al., 2015).

We identified 4 of the 24 *CYP2B6* variants listed by the PGKB, only rs4803419 (Level 2B) showed differential allele frequencies Natives > Mestizos > MXL > CEU, suggesting that Natives and Mestizos are 2× more likely to have decreased CYP2B6 metabolism due to rs4803419 which is in LD with rs3745274 a Level 1 variant. This is mostly relevant for efavirenz elimination, since CYP2B6 is its major metabolizing enzyme. A recently published guideline considers rs3745274 as the pharmacogenetic marker for efavirenz dosing assessment (Desta et al., 2019).

We identified five variants with lower allele frequency in Natives vs. Mestizos, CEU or MXL on, CYP2C19*2 rs4244285, *NAT2 rs1799930*, *CYP2D6* rs28371725, *SLCO1B1* rs41419015, and *ABCB1* rs2032582. In addition to the already discussed VKORC1–CYP2C9*2/*3 interplay, our results on *CYP2C9* validate previous reports showing that Mestizos are 3× and 4× more likely to show impaired CYP2C9 metabolism than Natives due to CYP2C9*2 and *3 (Villegas-Torres et al., 2015). CYP2C9 is the second most expressed CYP in liver after CYP3A4, and impacts 15% of all drugs including losartan, NSAIDs, phenytoin, and hypoglycemic agents, (Sconce et al., 2005;Van Booven et al., 2010). Here, we hypothesize that Natives will show a lower probability of impaired CYP2C9 activity compared to Mestizos and both of them lower to CEU. These comparisons have been previously confirmed for *CYP2C9*, *ABCB1*, *CYP3A5*, and *CYP2C19* (Vargas-Alarcón et al., 2014; Zhou et al., 2017). For *CYP2C19*, we can infer that a higher proportion of Natives are normal/extensive metabolizers. Clopidrogrel dosing guideline lists around 40 variants on *CYP2C19*, we identified 3 of the 10 variants reported for the Americas, CYP2C19*3 and *4 were rare (<1%) in Mestizos and CEU, and we did not observe them in Natives. The CPIC reported these variants associated to ADRs and are relevant for dose estimation for clopidogrel, escitalopram, and voriconazole. It is possible that ADR or dose adjustments would be seen in a lower proportion in Mexicans. Similarly, for *SLCO1B1* rs4149015 and *ABCB1* rs2032582 frequencies were lower in Natives, from we could infer a higher efficacy of drugs such as statins, atazanavir, or sunitinib.

Similarities among Natives, Mestizos, MXL, and CEU were observed for several variants on *UGT1A1*, *DPYD*, *ABCB1*, *CBR3*, *CYP2B6*, *COMT*, and *TPMT* hinting towards a comparable pharmacokinetics between populations for the role variants observed. Maybe these variants could smoothly transition into clinical implementation, but these inferences should not only be validated, but should also account for unreported, rare, and novel variation to determine the overall pharmacogenetic impact for each gene-drug combination.

### Novel Variants on PGKB Genes

**Figure 2:**
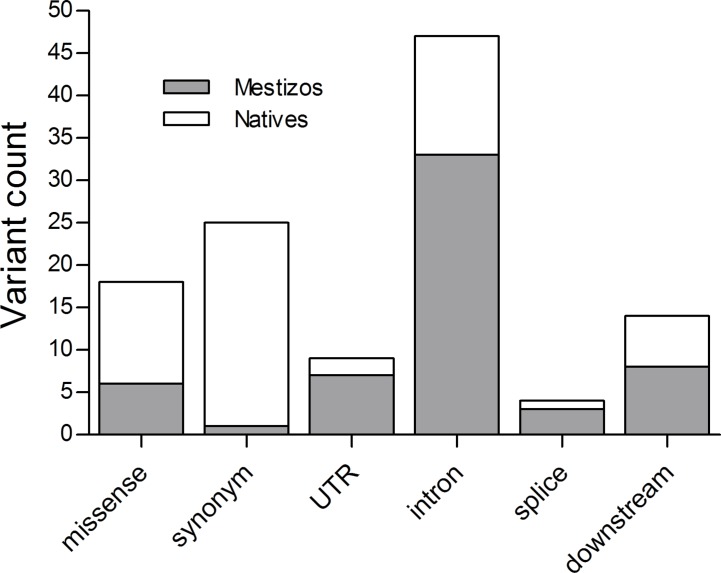
Functional impact of novel variation in Mestizos (gray) and Natives (blank).

Pharmacogenetic research in populations from Mexico has been actively increasing, however the number of publications by 2018 barely reached 0.3% of the >22,000 NCBI pharmacogenetic/pharmacogenomics reports. Here, we identified novel variants on 16 of the 17 studied PGKB genes, in Mestizos (58 variants) and Natives (67 variants, **Figures 1** and **2**, **Table 3** and **Supplemental Table 2**). For some genes novel variants were identified only in Natives, *APOE* (1), *UGT1A1* (3), and *VKORC1* (3) or only in Mestizos, *COMT* (4), *CYP2B6* (1), *CYP2D6* (3), and *CYP3A4* (6). Although these counts are influenced by sample size, it may offer an estimate of the completion in gene variation. Interesting differences in the number of novel variants arose for *ABCB1*, *ACE*, *DPYD*, *UGT1A1*, and *VKORC1*. For example, the transporter gene *ABCB1* showed 25 novel variants in Natives, and only 5 in Mestizos, the functional impact of most of these were confirmed by independent algorithms and 10 were validated as deleterious. Similar results were observed for variants on *DPYD* suggesting that variation on these genes is far from complete in Natives. In the previous section, we mentioned that the allele frequency of several PGKB variants in Natives were similar to that in other populations however, novel variation indicates that it is likely that the overall pharmacogenetic impact has not been fully described. This is in agreement with a few reports on *DPYD* and *ABCB1* novel variation in underrepresented populations, indicating that our current information of pharmacogenetic predictors remains to be thoroughly depicted (Mukonzo et al., 2009; Del Re et al., 2015; Elraiyah et al., 2017).

Variation on *CYP2C19* did not show novel variants in Natives, we may infer that most common variation has been recorded for this gene, and that further phenotypic variability may be imputed to rare mutations. In Mestizos, we found 10 novel variants, 2 were missense deleterious (POS.10:96522531 and POS 10:96609809) which may be relevant for dozens of drugs including, proton pump inhibitors, antiepileptics, antiplatelets, and antidepressants. We anticipated fewer novel variation on this gene since intense research has been done given its high importance in multiple drug-drug interactions and drug metabolism. Nevertheless, 10 novel variants were identified in Mestizos, which may accord with a couple of recent NGS studies, reporting novel, common, and rare variation on this gene in Asians and Africans (Matimba et al., 2009; Han et al., 2017; Dai et al., 2015).

Interestingly, we detected 14 novel variants with a MAF>3% on (4 SNVs MAF:8%), *DPYD* (1 SNV, MAF:3%), *CYP2C9* (1 SNV, 3%), *ACE* (1 SNV, MAF:5%), *APOE* (1 SNV, MAF:5%), *CYP3A4*/5 (5 SNVs MAF 3%), and *CYP2D6* (1 SNV, MAF:40%), although none of these were predicted as deleterious (**Supplemental Table 1**).

We are aware of the different sequencing techniques and size of the study, potentially affecting the number of variants and the inferences made. Also, PGKB variants not reported here reflect either lack of sequencing coverage or an allele frequency lower than 1%. Nevertheless, several of our observations have been confirmed by previous reports (Villegas-Torres et al., 2015) or paralleled those from Latin American countries such as Brazil with which we share pharmacogenetic similarities and differences ex. MAF for *CYP2B6* rs3745274, *CYP3A5* rs776746, *VKORC1* rs8050894, and *ABCB1* rs2032582, supporting the notion that pharmacogenetic diversity across the Americas ought to be consolidated.

## Conclusions

Our observations summarize variation in 55 pharmacogenes in 1,378 Natives and Mestizos from Mexico, focusing on 17 PGKB genes. This is one of the largest collections of genetic variability related to pharmacogenomics in Mexicans. Our report offers a collection of variants in core pharmacogenes, confirming previous knowledge and contributing to the list of novel variants that can be further investigated and may become a part of a preliminary catalogue for PK/PD, and phenotype-genotype correlations. These results may also complement genotyping platforms with relevant pharmacogenetic variants with specific population background. Future studies will seek to validate this variation and to confirm its potential application in pharmacogenomics.

## Data Availability Statement

The datasets analyzed for this study can be found at the databases: dbGAP: WES (Mestizos=968) (Flannick et al. 2019) [https://www.ncbi.nlm.nih.gov/gap/ phs001393 and phs001099], and at database, European Variation Archive (EVA) under project PRJEB343334 for: NGS-targeted 1 (Mestizos =110) (Gonzalez-Covarrubias et al. 2017) [EVA INMEGEN-ACOAG analysis: ERZ1079024], NGS-targeted 12 (Mestizos =146) (Gonzalez-Covarrubias et al. 2016) [INMEGEN-SCZ analysis: ERZ1079026], and NGS-targeted 3 128 (Mestizos = 60) (Cruz-Correa et al. 2017) [INMEGEN-ATV analysis: ERZ1079025]. WGS (Natives=94) Access to this dataset can be requested via email to Dr. Cristobal Fresno of 100G-Consortium, INMEGEN [cfresno@inmegen.gob.mx].

## Ethics Statement

The studies involving human participants were reviewed and approved by Comite de Etica e Investigacion, Instituto Nacional de Medicina Genomica #25/2016/I. The patients/participants provided their written informed consent to participate in this study.

## Author Contributions

VG-C conceived the analyses and wrote the paper. MM-F performed bioinformatics and statistical analyses. OC-C contributed to data collection. AM-H, HG-O, and FB-O contributed to patient recruitment and genetic experiments. AG-M contributed to patient recruitment and genetic experiments. JM-M recruited patients and performed genetic experiments. HN designed project, contributed to patient recruitment. LO conceived, coordinated, and executed the project. XS conceived and coordinated the project.

## Funding

Funding from Conacyt grant no. 252952 to XS, Conacyt grant no. 233970 to LO, Conacyt grant no. 272795 to VG-C and grant INMEGEN-No.14/2014/I.

## Conflict of Interest

The authors declare that the research was conducted in the absence of any commercial or financial relationships that could be construed as a potential conflict of interest.

## Abbreviations

NGS, next generation sequencing; WGS, whole genome sequencing; WES, whole exome sequencing; MAF, minor allele frequency; PGKB, Pharmacogenomics Knowledge Base; SNVs, single nucleotide variant; PK/PD, pharmacokinetics/pharmacodynamics.
