# Supplementary material for: Variation in Actionable Pharmacogenetic Markers in Natives and Mestizos From Mexico
Source: Front Pharmacol. 2019 Oct 10;10:1169. doi: 10.3389/fphar.2019.01169 (PMC6796793; doi:10.3389/fphar.2019.01169)
Supplement: Supplementary file 1 [file Table_1.pdf]

Supplemental T1. Novel variation in 17 PGKB genes

Gene	POS	Change	unctional impa	eterious Valid	MAF %
APOE	19:45412411	G/C	synonymous	0/3	10.6
ACE	17:61573984	C/T	coding	0/3	2.5
ACE	17:61575123	C/T	regulatory	0/3	1.06
ACE	17:61585438	A/G	coding	0/3	1.15
ACE	17:61585440	C/G	coding	0/3	1.16
ACE	17:61585444	G/C	coding	0/3	1.19
ACE	17:61587556	GA/AA	coding	0/3	5
ACE	17:61597227	T/C	transcript	1/3	1.06
ACE	17:61597264	G/T	transcript	0/3	2.92
ABCB1	7:87133076	G/C	downstream	0/3	1.09
ABCB1	7:87138330	C/T	transcript	0/3	2.5
**ABCB1**	**7:87165002**	**C/A**	**transcript**	**2/3**	**1.85**
ABCB1	7:87180070	C/A	missense	1/3	1.67
ABCB1	7:87196213	C/A	missense	1/3	1.82
ABCB1	7:87144590	A/G	missense	1/3	1.06
**ABCB1**	**7:87160718**	**C/A**	**synonymous**	**2/3**	**1.6**
ABCB1	7:87160733	A/G	synonymous	1/3	1.6
ABCB1	7:87160742	G/T	missense	1/3	1.6
ABCB1	7:87160744	A/G	missense	1/3	1.6
ABCB1	7:87160751	A/G	synonymous	1/3	1.6
ABCB1	7:87160754	T/G	synonymous	0/3	1.6
ABCB1	7:87160785	G/A	missense	1/3	1.06
**ABCB1**	**7:87160786**	**T/A**	**missense**	**2/3**	**1.06**
ABCB1	7:87175224	T/C	synonymous	0/3	1.06
ABCB1	7:87175258	T/C	missense	0/3	1.06
ABCB1	7:87175302	A/G	synonymous	1/3	1.06
ABCB1	7:87175329	A/G	synonymous	1/3	1.06
ABCB1	7:87175334	T/C	missense	1/3	1.06
ABCB1	7:87175338	G/A	splice	1/3	1.06
ABCB1	7:87179245	A/T	synonymous	1/3	1.06
ABCB1	7:87179263	T/C	synonymous	1/3	1.06
ABCB1	7:87179269	T/G	synonymous	1/3	1.06
**ABCB1**	**7:87179286**	**A/G**	**synonymous**	**2/3**	**1.06**
ABCB1	7:87179287	T/C	synonymous	1/3	1.06
ABCB1	7:87196186	T/C	missense	1/3	1.06
ABCB1	7:87196190	A/G	synonymous	1/3	1.06
ABCB1	7:87196193	T/C	synonymous	1/3	1.06
ABCB1	7:87196202	T/C	synonymous	1/3	1.06
ABCB1	7:87196238	A/G	synonymous	1/3	1.06
CBR3	21:37507219	C/A	UTR	0/3	1
COMT	22:19949754	A/T	intron	1/3	2.86

COMT	22:19954930	C/A	intron	0/3	1.89
COMT	22:19958910	C/A	downstream	0/3	1.28
COMT	22:19958912	C/A	downstream	0/3	1.89
CYP2B6	19:41497360	C/A	synonymous	0/3	1.69
CYP2C19	10:96454505	T/G	intron	0/3	2.7
CYP2C19	10:96479897	A/C	intron	0/3	1.12
CYP2C19	10:96479899	T/C	intron	0/3	1.15
CYP2C19	10:96522442	T/A	UTR	0/3	1.67
CYP2C19	10:96522531	C/A	**missense**	0/3	2.94
CYP2C19	10:96522710	A/C	intron	0/3	1.06
CYP2C19	10:96535790	C/A	intron	0/3	1.54
CYP2C19	10:96540083	T/G	intron	0/3	1.49
**CYP2C19**	**10:96609809**	**T/A**	**missense**	**2/3**	**1.15**
CYP2C19	10:96612219	T/C	intron	0/3	1.82
CYP2C19	10:96612984	C/A	UTR	0/3	2.06
CYP2C9	10:96702594	C/A	intron	0/3	3.01
CYP2C9	10:96703117	A/G	intron	0/3	1.06
CYP2C9	10:96705222	G/A	intron	0/3	1.06
CYP2C9	10:96748329	T/C	intron	0/3	2.88
CYP2C9	10:96749251	G/C	downstream	0/3	1.09
CYP2D6	22:42522759	G/C	splice	0/3	1.72
CYP2D6	22:42524143	A/C	intron	0/3	1.36
CYP2D6	22:42526061	T/G	intron	0/3	**40.91**
CYP3A4	7:99355212	G/A	downstream	0/3	1.39
CYP3A4	7:99365768	G/C	intron	0/3	3.28
CYP3A4	7:99365770	G/A	intron	0/3	3.28
CYP3A4	7:99366179	C/A	intron	0/3	1.47
**CYP3A4**	**7:99377662**	**C/A**	**missense**	**2/3**	**1.47**
CYP3A4	7:99381031	A/G	intron	0/3	2.5
CYP3A5	7:99245888	C/A	UTR	0/3	1.76
CYP3A5	7:99245891	C/A	UTR	0/3	1.86
CYP3A5	7:99248025	A/G	intron	0/3	1.06
CYP3A5	7:99257879	A/T	intron	0/3	2.13
CYP3A5	7:99260925	C/A	intron	0/3	3.82
CYP3A5	7:99263091	G/A	intron	0/3	2.38
CYP3A5	7:99264910	T/C	intron	0/3	3.51
CYP3A5	7:99264917	C/A	intron	0/3	3.51
CYP3A5	7:99277442	T/C	splice	1/3	1.95
DPD	1:97602855	G/T	intron	1/3	1.06
DPD	1:97915621	T/C	synonymous	1/3	1.06
DPD	1:97915642	A/G	synonymous	1/3	1.6
DPD	1:97915651	T/G	missense	1/3	1.6
DPD	1:98015187	A/T	missense	0/3	1.06
DPD	1:98015191	A/C	synonymous	1/3	1.06
DPD	1:98015212	T/C	synonymous	1/3	1.06
DPD	1:98015220	C/G	missense	1/3	1.06
DPD	1:98015227	A/G	synonymous	1/3	1.06
DPD	1:98015236	A/G	synonymous	1/3	1.06
DPD	1:98058901	A/G	missense	0/3	1.06
DPD	1:98058908	G/T	synonymous	1/3	1.6

DPD	1:98059027	G/C	intron	1/3	1.06
DPD	1:98059057	A/G	intron	1/3	1.06
DPD	1:98186061	C/T	UTR	1/3	1.69
DPD	1:98186277	C/T	UTR	1/3	1.06
DPD	1:98348549	T/C	intron	0/3	1.67
DPD	1:98348873	C/A	missense	1/3	1.67
DPD	1:98386502	T/A	UTR	0/3	3.06
NAT1/2	8:18070030	A/G	intron	0/3	1.06
NAT1/2	8:18070394	A/G	intron	1/3	1.06
NAT1/2	8:18081208	T/C	downstream	0/3	1.67
NAT1/2	8:18248089	T/C	downstream	0/3	1.67
TPMT	6:18139708	A/T	intron	0/3	1.64
TPMT	6:18139709	A/G	intron	0/3	1.61
TPMT	6:18143966	A/G	splice	0/3	8.33
TPMT	6:18143968	A/G	intron	0/3	8.33
TPMT	6:18143970	T/C	intron	0/3	8.33
TPMT	6:18143973	T/C	intron	0/3	8.33
TPMT	6:18148356	T/C	intron	0/3	1.06
TPMT	6:18155178	T/A	downstream	0/3	1.06
UGT1A1	2:234544998	G/T	downstream	0/3	1.6
UGT1A1	2:234638262	T/A	missense	1/3	1.06
UGT1A1	2:234669746	T/C	synonymous	1/3	1.06
VKORC1	16:31099858	C/A	intron	0/3	2.66
VKORC1	16:31105136	T/G	downstream	0/3	1.06
VKORC1	16:31110460	G/T	downstream	1/3	1.06
