# Supplementary material for: Variation in Actionable Pharmacogenetic Markers in Natives and Mestizos From Mexico
Source: Front Pharmacol. 2019 Oct 10;10:1169. doi: 10.3389/fphar.2019.01169 (PMC6796793; doi:10.3389/fphar.2019.01169)
Supplement: Supplementary file 2 [file Table_2.pdf]

ST2. Novel variation in PGKB genes in Natives and Mestizos

[1]No variants with MAF>1% were observed on *SLCO1B1*

Gene	Natives mean, MAF%	Mestizos mean, MAF%	Observations for novel variants
ABCB1	25 (1-1.6%), 8 missense, 16 syn, 1 spliceregion	5 (1-2.5%), 2 intron, 2 missense, 1 downstream	Natives. 20 variant's functional impact validated in more than one algorithm, 3 missensevariant's functional impact validated in more than 2 algorithms. Mestizos: one variant's functional impact validated in more than one algorithm and in at least 2 algorithms. 10 deleterious
ACE	2 (1%), downstream	6 (1-3%), 5 intron, 1 downstream	In Natives one variant's functional impact validated in more than one algorithm 8 deleterious
APOE	1 (1%), syn	0	None deleterious
CBR3	1 (1%) UTR	1 (0.5%), UTR	Variant observed in both populations None deleterious
COMT	0	4 (1-3%), 2 intron, 2 downstream	One variant's functional impact validated in more than one algorithm, 2 deleterious
CYP2B6	0	1 (1.7%), syn	None deleterious
CYP2C19	1 (1.1%), intron	10 (1-3%), 6 intron, 2 UTR, 2 missense	Variant's 10:96609809 functional impact validated in more than one algorithm, 2 deleterious
CYP2C9	2 (1.1%), intron	3 (1-3%), 2 intron, 1 downstream	None deleterious
CYP2D6	0	3 (1-40%), 2 intron, 1 spliceregion	2 deleterious
CYP3A4	0	6 (1-3%), 4 intron, 1 missense, 1 downstream	Variant's 7:99377662 functional impact was validated in more than one algorithm in Mestizos, 1 deleterious
CYP3A5	2 (1-2%), intron	7(1-4%), 4 intron, 2UTR, 1 spliceregion	Variant's 7:99277442 functional impact was validated in more than one algorithm in Mestizos, 1 deleterious
DPD	15 (1-1.6%), 3 intron, 4 missense, 7 syn, 1 UTR	4 (1-3%), 1 intron, 2 UTR, 1 missense	13 variant's functional impact were validated in more than one algorithm in Natives and 2 variant's in Mestizos, 5 deleterious
NAT2	2 (1%), intron	2 (1-2%), 2 downstream	Variant's 8:180070394 functional impact was validated in more than one algorithm in Native None deleterious
TPMT	2 (1%), 1 intron, 1 downstream	6 (1-8%), 5 intron, 1 spliceregion	None deleterious
UGT1A1	3 (1 -1.6%), 2 intron, 1 downstream	0	2 variant's functional impact were validated in more than one algorithm in Natives 2 deleterious
VKORC1	3 (1-3%), 1 intron, 2 downstream	0	Variant's 16:3111046 functional impact was validated in more than one algorithm in Natives 2 deleterious
